# Chondroitin sulfate proteoglycan 4 expression in chondrosarcoma: A potential target for antibody-based immunotherapy

**DOI:** 10.3389/fonc.2022.939166

**Published:** 2022-08-30

**Authors:** Sjoerd P. F. T. Nota, David O. Osei-Hwedieh, David L. Drum, Xinhui Wang, Francesco Sabbatino, Soldano Ferrone, Joseph H. Schwab

**Affiliations:** ^1^ Department of Orthopaedic Surgery, Massachusetts General Hospital, Harvard Medical School, Boston, MA, United States; ^2^ Section of Orthopaedic Oncology, Massachusetts General Hospital, Harvard Medical School, Boston, MA, United States; ^3^ Department of Orthopaedic Surgery, Amsterdam UMC, University of Amsterdam, Amsterdam, Netherlands; ^4^ Department of Surgery, Massachusetts General Hospital, Harvard Medical School, Boston, MA, United States

**Keywords:** chondrosarcoma, CSPG4, immunotherapy, CAR T, dedifferentiated chondrosarcoma

## Abstract

Chondrosarcoma is a common primary bone malignancy whose phenotype increases with its histologic grade. They are relatively resistant to chemotherapy and radiation therapy limiting curative options for disseminated disease. Chondroitin sulfate proteoglycan 4 (CSPG4) is a cell surface proteoglycan that is highly expressed across various human cancers, including chondrosarcoma, and has restricted distribution in healthy tissues, making it an attractive target for the antibody-based therapy. CSPG4 specific chimeric antigen receptor (CAR) T cell therapies have been shown to be effective in treating other cancers such as melanoma and triple negative breast cancer. The goal of this study was to assess the prevalence of CSPG4 in human chondrosarcoma and to assess the efficacy of CSPG4 specific CAR T cells in lysing chondrosarcoma cells *in vitro*. Using immunohistochemistry (IHC), we stained a tissue microarray containing primary conventional and dedifferentiated chondrosarcoma from 76 patients with CSPG4 specific monoclonal antibodies (mAbs). In addition, we incubated 2 chondrosarcoma cell lines with CSPG4-targeting CAR T cells and subsequently evaluated cell survival. Our results showed medium to high expression of CSPG4 in 29 of 41 (71%) conventional chondrosarcoma tumors and in 3 of 20 (15%) dedifferentiated chondrosarcoma tumors. CSPG4 expression showed a positive association with time to metastasis and survival in both subtypes. CSPG4 CAR T treated cell lines showed a lysis of respectively >80% and 70% demonstrating CSPG4-targeted CAR T cells effective in killing CSPG4-positive chondrosarcoma tumors.

## Introduction

Chondrosarcoma is the second most common primary malignant cancer of the bone in which the tumor behavior and the histologic grade are closely associated (1, 2). Low-grade chondrosarcoma are slow growing, may be locally aggressive, but carry a low risk of metastasizing. High-grade chondrosarcoma and dedifferentiated chondrosarcoma are aggressive and carry a high risk of early metastasis and recurrence (3, 4). The standard of care for chondrosarcoma remains complete surgical excision. There exists a need for effective systemic therapies in the case of locally recurrent disease, metastasic disease and when surgical resection leads to unacceptable morbidity to the patient. Chemotherapy and radiotherapy have not been effective in salvaging these cases and their use is generally limited to palliative settings (5, 6).

In previous studies, our group and others have shown that HLA I expression is reduced in conventional chondrosarcoma, suggesting that immunotherapeutic strategies targeting machinery upstream of tumor antigen cross-presentation will be ineffective in the absence of induced HLA I stimulation (7–9). This contrasts with dedifferentiated chondrosarcoma where limited defects in HLA I expression were observed and therefore potentially benefit from checkpoint blockade inhibition, a treatment approach that utilizes functional HLA I antigen expression (8, 10).

Here, we explore chimeric antigen receptor (CAR) T cell therapy, a tumor killing mechanism that is independent of HLA-I antigen expression or functionality. CAR T cells express synthetic receptors that recognize and lyse cells expressing a specific target antigen. CAR binding to target antigens expressed on the cell surface is independent from the MHC receptors, yielding potent T cell activation and anti-tumor responses (11, 12). CAR T immunotherapy has been a remarkable success in leukemias with limited success in solid tumors (13–16), however, based on its MHC-independent activation of T cells, CAR T therapy represents a potential therapy in chondrosarcoma.

In this study, we focus on chondroitin sulfate proteoglycan 4 (CSPG4) as a potential immunotherapeutic target in chondrosarcoma based on the discovery that it is highly expressed in chemically-induced rat chondrosarcoma, and originally identified as rat chondroitin sulfate proteoglycan nerve-glial 2 (NG2), later to be shown to share 100% homology with human CSPG4 (17–19). Earlier we have described the CSPG4 expression in chondrosarcoma and chordoma comparing the genetic profile of chondrosarcoma with other cancers (20). CSPG4 is a cell surface proteoglycan that is highly expressed across various types of human cancers with restricted expression in healthy tissues, making it an attractive target for antibody-based cancer immunotherapy. Importantly, CSPG4 specific monoclonal antibodies and adoptive cell transfer therapies targeting CSPG4 have been effective in pre-clinical models by inhibition tumor growth and proliferation, making it a promising target for clinical application (18, 21–24). The clinical relevance of CSPG4 has been demonstrated in recent studies showing that this antigen is an independent risk factor for decreased survival in epithelial ovarian cancer (25).

The immunotherapeutic significance of CSPG4 in chondrosarcoma has not yet been explored and therefore we evaluated its efficacy as a potential target in chondrosarcoma immunotherapy by assessing the expression of CSPG4 in a large series of surgically removed conventional and dedifferentiated chondrosarcoma. We also investigated the association of CSPG4 expression with the overall survival and the development of metastases. In addition we investigated the effectiveness of CSPG4-specific CAR T cell therapy in eliminating chondrosarcoma cells in 2 chondrosarcoma cell lines.

## Materials and methods

### Clinical chondrosarcoma samples

We utilized the Orthopaedic Oncology registry at the Massachusetts General Hospital, Boston, Massachusetts to identify chondrosarcoma cases treated surgically from 1993 to 2013. We selected patient samples with a conventional or dedifferentiated chondrosarcoma in a non-cranial location tumor who had the primary tumor surgically removed at our hospital (IRB Approval #*2013P001012*).

### Inclusion criteria

A two-year minimum clinical follow-up time or death was set for inclusion. Based on this criterion, we selected 82 subjects for further studies. Six of the 82 patient samples were excluded because of insufficient amount of paraffin embedded tumor tissue for the study. The sample size for our study was 76; 52 cases of diagnosed conventional chondrosarcoma ranging from grade 1-3 and 24 cases of dedifferentiated chondrosarcoma. The average age for the 76 patients in the cohort was 56 ± 14 years (range, 18-83) and 53% of these patients were male. The mean age for the grade 1, grade 2 and grade 3 patients were respectively 49 ± 14 years (range, 18-69), 57 ± 16 years (range, 33-83) and 56 ± 13 years (range, 39-73). The average age of patients with a dedifferentiated chondrosarcoma was 59 ± 13 years (range, 39-82) (**Table 1**).

**Table 1:** Chondrosarcoma-clinical and pathological characteristics, n = 76.

Grade 1		n = 17	Grade 2		n=30
Age, years	Mean ± SD	range	Age, years	Mean ± SD	range
n = 17	49 ± 14	18-69	n = 30	57 ± 16	33–83
**Sex**	n	%	**Sex**	n	%
male	6	35	male	15	50
female	11	65	female	15	50
**Metastasis at presentation**	n	%	**Metastasis at presentation**	n	%
yes	0	0	yes	0	0
no	17	100	no	30	100
**Metastasis**	n	%	**Metastasis**	n	%
yes	0	0	yes	6	20
no	17	100	no	24	80
**Grade 3**		**n = 5**	**Dediff. CS.**		**n = 24**
**Age, years**	**Mean ± SD**	**range**	**Age**, years	**Mean ± SD**	**range**
n = 5	56 ± 13	39-73	n = 24	59 ± 13	39-82
**Sex**	n	%	**Sex**	n	%
male	3	60	male	15	63
female	2	40	female	9	38
**Metastasis at presentation**	n	%	**Metastasis at presentation**	n	%
yes	0	0	yes	7	29
no	5	100	no	17	71
**Metastasis**	n	%	**Metastasis**	n	%
yes	2	40	yes	22	92
no	3	60	no	2	8.3

### Tissue microarray

Tissue blocks matched with hematoxylin and eosin-stained slides were obtained from the Department of Pathology, reviewed by 2 investigators (SN, JS) and independently confirmed by a senior musculoskeletal pathologist for adequate tissue. Areas to be sampled were circled with a fine tipped felt pen. Multiple circles were made per sample to account for tissue heterogeneity. Circled areas on the slide were compared to the corresponding paraffin block to identify areas to be included in the tissue microarray (TMA) prior to assembly where 4 mm cylinders of representative regions were excised from each block. Eight primary-met pairs were included in the TMA representing 3 cases of conventional chondrosarcoma and 5 cases of dedifferentiated chondrosarcoma.

Eight enchondroma samples as well as clinical cartilage, spleen, liver and lymph node tissue were included as negative controls. Clinical melanoma metastasis sample, murine melanoma xenograft and liver samples were included as positive controls. Control samples were included to confirm staining specificity of monoclonal antibody (mAb) D2.8.5-C4B8. Tissue samples were placed randomly and individually arranged into a total of 6 paraffin blocks.

### CSPG4 monoclonal antibody

The CSPG4-specific mAb D2.8.5-C4B8, a mouse IgG1, is secreted by a hybridoma generated from a BALB/c mouse immunized with a peptide corresponding to the amino acid sequence. The mAb was purified from ascitic fluid by affinity chromatography on a Protein G column (GE Healthcare Life Sciences, Pittsburgh, PA). The mAb preparation was controlled for purity and activity by SDS-PAGE and by binding assays with the cognate antigen. The mAb D2.8.5-C4B8 has been shown to recognize an epitope of CSPG4 in formalin-fixed paraffin-embedded (FFPE) tissue sections (24). Cytoplasmic membrane staining was considered positive for CSPG4.

### Immunohistochemical staining of CSPG4 protein

The immunohistochemical staining of CSPG4 protein was performed as described before (18). Following the deparaffinization and rehydration of the tumor tissue, antigen retrieval was achieved by indirectly heating the tissue in a 1mM EDTA buffer with a pH of 8.0 for 15 minutes. To prevent non-specific binding we incubated the tissue microarray slide with a blocking buffer containing 1% Bovine Serum Albumin (BSA) and 5% normal horse serum (NHS) in Tris-Buffered Saline with Tween 20 (TBST). Incubation of the slides was performed overnight at 4 degree Celsius with the CSPG4-specific mAb D2.8.5-C4B8 (3 μg/mL) diluted in 1% BSA and 5% NHS in TBST. Subsequent staining was achieved with the DAKO EnVision+ System-HRP (Dako North America, Inc.) and substrate diaminobenzidine (Dako North America, Inc.). Counterstaining was performed with Mayer’s Hematoxylin (Lillie’s Modification, Dako North America, Inc.). Finally we mounted the slides with Depex mounting medium (Electron Microscopy Sciences ^®^).

The staining intensity was graded by semiquantitative analysis by 2 investigators (SN, FS) where CSPG4 staining using IHC was scored using a categorical classification, 1-low expression; 2-medium expression; 3-high expression.

### Generation of CAR-T cells

CAR-T cells were generated as described before (26) Peripheral blood mononuclear cells (PBMCs) were isolated from healthy human donor blood (Research Blood Components, Cambridge, MA) with Lymphoprep (Stem Cell Technologies, Cambridge, MA). On day 0, the PBMCs (1 * 10^6^/well) were activated in a nontreated 24-well cell culture plate (catalog 351147, Corning) precoated with 1 μg/mL CD3 (clone OKT3; Miltenyi Biotec) and 3 μg/mL CD28 antibody (clone CD28.2; BD Biosciences) in complete medium (45% RPMI1640 and 45% Click’s medium (Irvine Scientific), 10% FBS, 1% Penicillin, and 1% Streptomycin (Corning)). On day 1, activated T cells were expanded by addition of IL-7 (10 ng/mL, PeproTech, Cranbury, NJ) and IL-15 (5 ng/mL, PeproTech; CAR T medium). On day 2, activated and T cells were transferred to a 24-well plate coated with RetroNectin (Takara Bio Inc., Ann Arbor, MI) containing retroviral particles of either CSPG4, or CD19 CAR construct. Following a 48 h incubation (day 4), transduced T cells were transferred to tissue culture-treated 24-well plates (# 353047; Corning) with each well containing 0.5 mL of the activated T-cell suspension (5 *10^5^ cells/well) and 1.5 mL of fresh CAR-T medium. On day 6, an aliquot of transduced cells was analyzed for transduction efficiency, CAR-T cells in suspension were spun down, 50% supernatant was replaced with the fresh medium 50: 50 (v/v) old medium: new medium. On day 8, CAR T cells were re-suspended in 2 mL of fresh CAR-T medium at 1 * 10^6/^well to further expand cells. On day 10, cells in suspension were spun down, 50% supernatant was replaced with the fresh medium. On day 13 to 14, CAR T cells and nontransduced T (NT) cells grown at similar conditions were collected, aliquoted, and frozen for storage in a liquid nitrogen freezer for the experiments.

### *In vitro* cell cytotoxicity assays

CSPG4 CAR T cells and target cells were co-cultured at indicated E:T ratios for 24 hrs. CAR T cells in the cell suspension were removed, and the viability of adherent target cells was quantitated by MTT assays. Mean ± SEM of cell lysis (%) of different cell populations in the chondrosarcoma cell line CS1 and SW1353 are shown. The human melanoma cell line M14 which does not express CSPG4 and M14/CSPG4 which expresses CSPG4 after stably transfected with CSPG4 plasmid DNA were used as specificity controls (27). CD19 CAR T cells were also used as a negative control since none of the target cells express CD19. The experiments were performed in triplicate and repeated 3 times, ***p<0.001.

### Statistical analysis

Comparisons between groups were performed using Fisher’s exact test. For bivariate analysis we used the log-rank test of equality across strata. This was used both for binary and categorical variables to identify factors that impact overall survival and the time to metastasis. For continuous variables as well as for binary/categorical variables we used a Cox regression analysis. Differences between CSPG4 CAR T cell-mediated cytotoxicity on different cell populations (including all E:T ratios) was detected using a chi-square test in a two-way ANOVA. STATA 12 software was used for statistical analysis purposes. *p*-values less than or equal to 0.05 were considered significant.

## Results

### Clinical outcomes for 76 patients with chondrosarcoma

The mean and median follow-up were 5.3 ± 4.5 years (range, 0.014-19) and 4.3 years (IQR, 1.2-8.1) respectively, for the entire cohort. The mean and median follow-up for the subjects who survived long term (n= 43) were 7.8 ± 4.2 years (range, 2.0-19) and 7.1 years (IQR, 4.2-9.3) respectively. In our cohort 39 patients died due their chondrosarcoma. All subjects diagnosed with chondrosarcoma grade 1 (n=17) disease remained alive throughout the duration of the study. Seven out of the 30 subjects diagnosed with chondrosarcoma grade 2 died from their disease [mean survival time: 4.8 ± 2.3 years (range, 0.68-8.3), median survival time: 4.8 years (IQR, 4.0-6.2)] and 2 out of 5 patients with chondrosarcoma grade 3 died from their chondrosarcoma (respectively 1.3 and 3.4 years after their pathologic diagnosis). All 24 patients with a dedifferentiated chondrosarcoma died from their disease (mean survival time: 1.2 ± 1.6 years (range, 0.013-6.6), median survival time: 0.71 years (IQR, 0.38-1.3).

### CSPG4 expression

**Figure 1:**
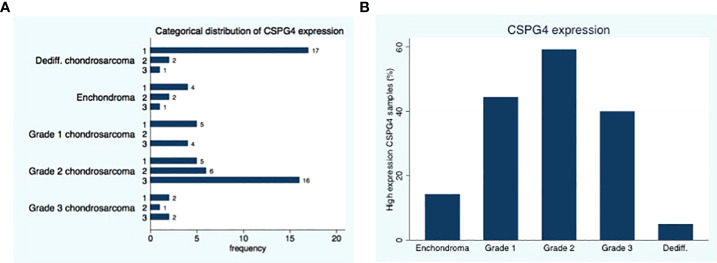
Distribution of CSPG4 Expression in Chondrosarcoma. **(A)** CSPG4 staining using IHC was scored by 2 investigators using a categorical classification, 1-low expression; 2-medium expression; 3 high-expression. **(B)** The number of samples within each category that stained positive with high intensity (categorical classification 3) was plotted as a percentage of the samples in the cohort; the distribution of high intensity CSPG4 positive samples showed a unimodal distribution with the highest percentage observed in grade II. Fisher’s exact p = 0.001.

Twenty-nine of the 41 (71%) conventional chondrosarcoma available for analysis show a positive (medium or high) CSPG4 expression. the largest percentage of positive samples in grade 2 (22 our of 27 tumors, 81%), followed by grade 3 (3 out of 5 tumors, 60%) and the lowest expression in grade 1 conventional chondrosarcomas (4 our of 9 tumors, 44%), (p=0.19, **Figure 1**).

In the dedifferentiated chondrosarcoma 3 out of 20 tumors, show positive (medium or high) CSPG4 expression (15%). The expression of CSPG4 in the dedifferentiated chondrosarcoma is less than the CSPG4 expression of conventional chondrosarcoma (P<0.001).

**Figure 2:**
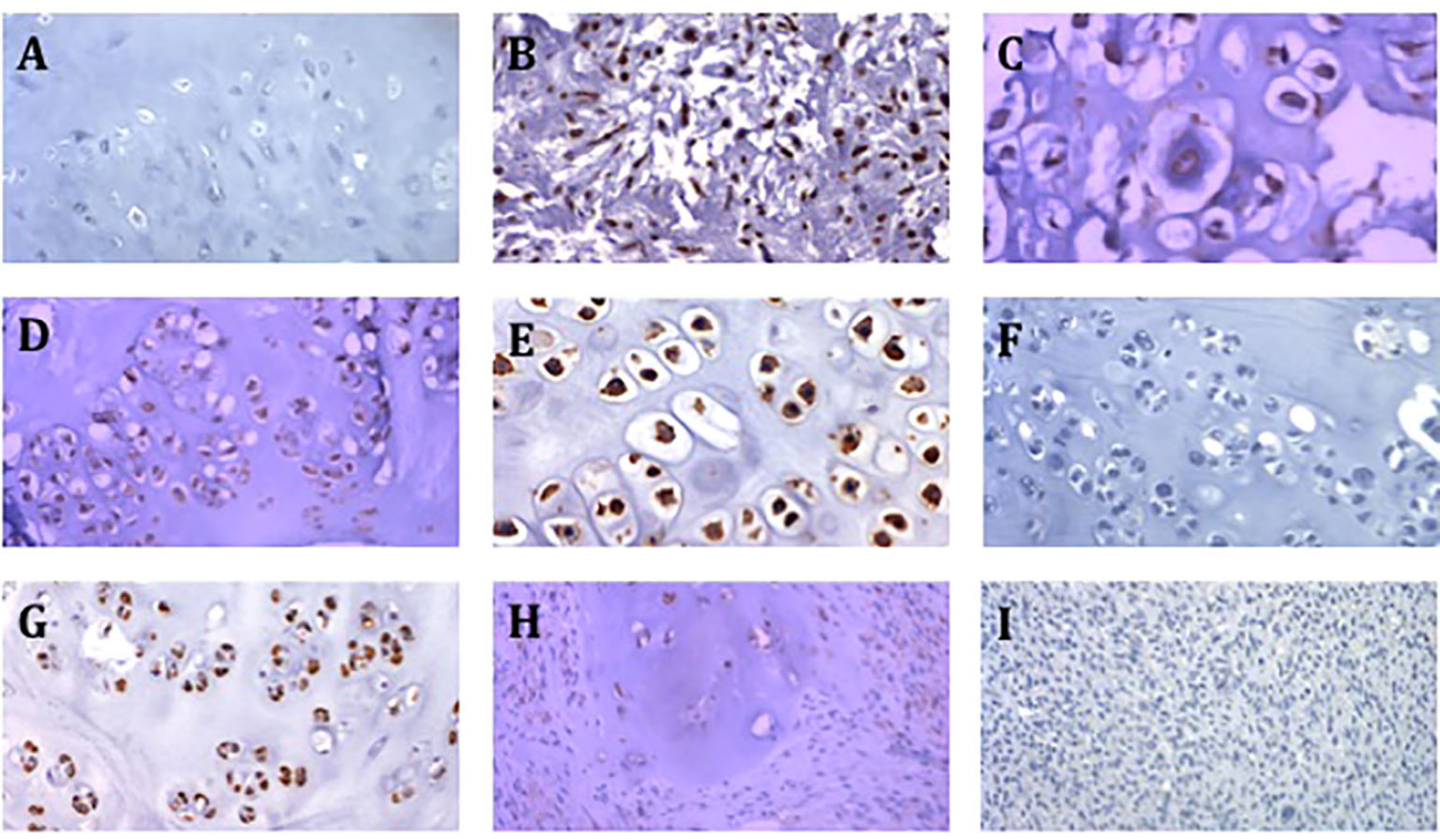
Representative images of CSPG4 staining of primary chondrosarcoma tumors. **(A)** CSPG4 negative stain in enchondroma (200x magnification). **(B)** CSPG4 positive stain in grade 2 chondrosarcoma (200x magnification). **(C)** CSPG4 positive stain in grade 2 chondrosarcoma (400x magnification). **(D)** CSPG4 positive stain in grade 2 chondrosarcoma (200x magnification). **(E)** CSPG4 positive stain in grade 2 chondrosarcoma (400x magnification). **(F)** CSPG4 negative stain in grade 2 chondrosarcoma (200x magnification). **(G)** CSPG4 positive stain in grade 2 chondrosarcoma (200x magnification). **(H)** CSPG4 positive stain in dedifferentiated chondrosarcoma (200x magnification). **(I)** CSPG4 negative stain in dedifferentiated chondrosarcoma (200x magnification).

Of the 3 conventional chondrosarcoma metastases, 2 showed a high expression of CSPG4 and 1 metastasis showed a medium expression. All 4 dedifferentiated chondrosarcoma metastasis showed a low CSPG4 expression. Three out of the 7 enchondroma we tested showed medium or high CSPG4 expression. Representative staining results of CSPG4 are presented in **Figure 2**.

### Conventional chondrosarcoma, survival analysis

**Figure 3:**
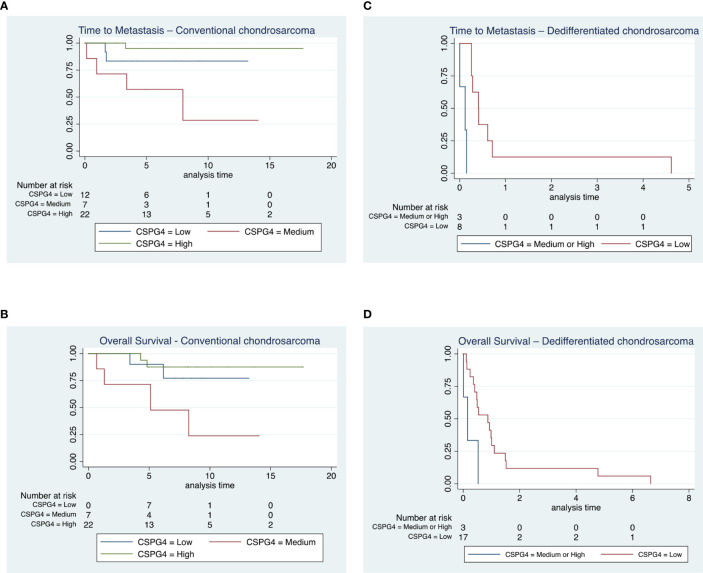
Time to metastasis and overall survival in conventional and dedifferentiated chondrosarcoma. **(A, B)** All patients diagnosed with chondrosarcoma grade 1 (n = 17) remained alive throughout the duration of the study, 7 out of the 30 subjects diagnosed with chondrosarcoma grade 2 and 2 out of 5 patients with chondrosarcoma grade 3 died from their disease. We observed a shorter time to metastasis in subjects with medium CSPG4 expression compared to the other groups in CSPG4 expression (p = 0.004) and there was a shorter time to death in subjects with a medium CSPG4 expression (p = 0.019). **(C, D)** All 24 patients with a dedifferentiated chondrosarcoma died from their disease. We observed a shorter time to metastasis in subjects who had medium and high CSPG4 expression when compared to subjects with low or no CSPG4 expression in chondrosarcoma (p = 0.048). In addition overall survival was shorter in subject who exhibited medium and high CSPG4 expression compared to subjects with low or no CSPG4 expression in chondrosarcoma tissues (p = 0.024).

Using bivariate analysis, we observed a shorter time to metastasis in subjects with medium CSPG4 expression compared to the other groups in CSPG4 expression (p=0.004, **Figure 3A**) and there was a shorter time to death in subjects with a medium CSPG4 expression (p=0.019, **Figure 3B**).

We did not observe any difference in time to metastasis (p=0.68) and in time to death (p=0.95) following surgical resection of primary tumor when we compared all CSPG4-positive samples regardless of rating to CSPG4-negative samples.

### Dedifferentiated chondrosarcoma, survival analysis

In the dedifferentiated chondrosarcoma cohort, we observed a shorter time to metastasis in subjects who had medium and high CSPG4 expression when compared to subjects with low or no CSPG4 expression in chondrosarcoma tissues, (p=0.048, **Figure 3C**). This difference was preserved when we compared all CSPG4 expressing samples (regardless of rating) with CSPG4-negative using bivariate analysis (p=0.0004).

Overall survival was shorter in subject who exhibited medium and high CSPG4 expression compared to subjects with low or no CSPG4 expression in chondrosarcoma tissues (p=0.024, **Figure 3D**). The 3 patients that have a medium or high CSPG4 expression have a shorter overall survival [mean survival time: 85 ± 97 days (range, 5-193), median survival time 57 days (IQR, 5-195)] compared to the 17 patients that show no or low CSPG4 expression [mean 475 ± 638 days (range 40-2427), median survival time 319 days (IQR, 148-402)].

### CSPG4 CAR T cells are effective in killing chondrosarcoma cells *in vitro*

**Figure 4:**
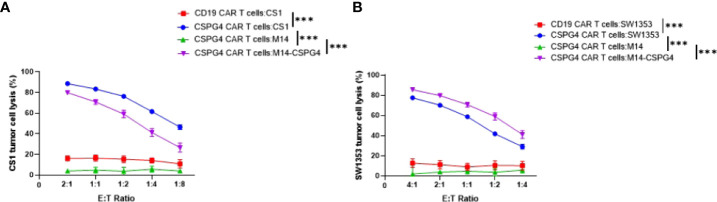
CSPG4-CART cells are effective in killing CSPG4-expressing chondrosarcoma cells. CSPG4 CAR T cells and target cells were co-cultured at indicated (E) T. ratios for 24 hrs. CAR T cells in the cell suspension were removed, and the viability of adherent target cells was quantitated by MTT assays. Mean ± SEM of cell lysis (%) of different cell populations in the chondrosarcoma cell line CS1 **(A)** and SW1353 **(B)** are shown. The human melanoma cell line Ml4 which does not express CSPG4 and M14/CSPG4 which express CSPG4 after stably transfected with CSPG4 plasmid DNA were used as specificity controls^25^. CD19 CAR T cells were also used as a negative control since none of the target cells express CD19. The experiments were performed in triplicate and repeated 3 times. Differences between CSPG4 CART cell-mediated cytotoxicity on different cell populations (including all E .T ratios) was detected using a chi square test in a two-way ANOVA. ***p < 0.001.

To test whether CSPG4 antigen is an effective target for the CAR T cell-mediated killing of chondrosarcoma cells, CS1 and SW1353 chondrosarcoma cells were incubated with CSPG4-targeting CAR T cells. Subsequently we evaluated the chondrosarcoma cell survival using the MTT assay. We observed dose-dependent increases of CS1 tumor lysis of >80% in CSPG4 CAR T cell treated cells at an E:T ratio of 1:1 versus <5% tumor lysis in the CD19 CAR T cell treated group. Similar dose-dependent increases in tumor lysis were observed in SW1353 chondrosarcoma cells, with 70% tumor lysis in CSPG4 CAR T cell treated cells at an E:T ratio of 1:1 versus <5% tumor lysis in the CD19 CAR T cell treated group (**Figure 4**). In both experiment set-ups there was no dose-dependent increases in M14 tumor cell killing with <2% tumor lysis at an E:T ratio of 1:1. When M14 cells were transfected to express CSPG4 antigen on its extracellular surface, a >30-fold increase in tumor lysis was observed in CSPG4-transfected M14 cells when treated with CSPG4 CAR T cells at a 1:1 E:T ratio (**Figure 4**).

## Discussion

The current study outlines CSPG4 expression in a large series of chondrosarcoma patients showing that medium to high CSPG4 expression is associated with shorter time to metastasis and reduced overall survival in conventional and in dedifferentiated chondrosarcoma. This finding is consistent with the association of CSPG4 and overall survival in other tumor types such as breast carcinoma, ovarium carcinoma and osteosarcoma (25, 28, 29). There appears to be a biphasic pattern where CSPG4 expression increases with histological grade from enchondroma up to grade 2 and begins to decrease in grade 3 and in dedifferentiated chondrosarcoma. We interpret this observation as a possible immune escape mechanism in more aggressive lesions where subjects whose adaptive immune system generated antibodies to CSPG4 lead to an evolutionary tumor response to downregulate this receptor. This theory is supported by findings of improved survival in melanoma patients who endogenously generated CSPG4-targeted antibodies (17, 30).

We have previously described the CSPG4 expression in chondrosarcoma and chordoma comparing the genetic profile of chondrosarcoma with other cancers (20). CSPG4 is a transmembrane proteoglycan that facilitates the interaction of cells with the extra-cellular matrix components: collagen types II, V, and VI, tenascin, laminin and fibronectin (17). CSPG4 allows cancer cells to proliferate and invade through the matrix leading to metastasis *via* downstream signaling pathways involving integrin-related signal transduction (31–33).

Chondrosarcoma is generally treated surgically with adequate surgical margins to control the tumor growth (34, 35). While surgery is the primary treatment of chondrosarcoma, its outcome is poor in higher-grade tumors, recurrent and metastatic tumors (35–37). Importantly, chondrosarcoma are relatively resistant to chemotherapy and radiation therapy limiting curative options for disseminated disease (5, 6).

Immunotherapy has been successfully applied clinically with many ongoing trials across many cancer types as well as various host immune machinery are currently being investigated in various passive and active immunotherapeutic protocols. One of the leading strategies is CAR T cell therapy where T cells are genetically engineered to express tumor specific antibody fragments to target and lyse cancer cells (38). Adoptive transfer of CAR T-cells has shown remarkable efficacy in treating mainly leukemias (39, 40). For solid tumors, high-antigen heterogeneity, poor tumor core infiltration, low density of tumor specific antigens as well as shared antigens between tumor and healthy cells, and an immunosuppressive tumor microenvironment pose significant challenges for CAR T cell efficacy and demonstrate the risk of on-target but off-tumor toxicities (39–41). However CSPG4 is an attractive target in cancer cells due to its well-defined role in tumor cell growth, invasion and metastasis, and its restricted expression in healthy tissue (29). Genetically engineered CSPG4-targeted CAR T cells have been shown to control tumor growth cells *in vitro* and *in vivo* in with different cell lines engrafted NSG mice (human melanoma, head and neck squamous cell carcinoma and breast carcinoma) and has been able to kill CSPG4 expressing glioblastoma cancer stem cells (42, 43).

Importantly, we observed efficient killing of CSPG4-expressing chondrosarcoma cells *in vitro* when incubated with CSPG4-targeted CAR T cells demonstrating that CSPG4-targeted CAR T cell immunotherapy may be an effective adjuvant therapy in CSPG4-positive conventional and dedifferentiated chondrosarcoma tumors. This strategy might be of particular relevance since our group and others have shown that HLA class I expression is frequently reduced in conventional chondrosarcoma, limiting immunotherapeutic strategies targeting machinery upstream of tumor antigen cross-presentation in the absence of induced HLA class I stimulation (7–9). In contrast, the CAR T cell therapy is independent of HLA class I antigen expression and might give therapeutic options in chondrosarcoma tumors with defective HLA class I expression.

Furthermore, we can increase CSPG4 expression in CS1 and SW1353 chondrosarcoma cells with one subclinical dose of irradiation (10 Gy) (**Supplemental Figure 1**) suggesting that CSPG4 CAR T efficacy may further be augmented when combined with irradiation.

Taken together, these findings support the role of CSPG4 as a promoter of disease and therefore as a clinically relevant target in patients with chondrosarcoma. The results of this study demonstrate that CSPG4 directed therapies may be applicable to chondrosarcomas with over 70% of patients in our cohort demonstrating moderate to high expression in conventional chondrosarcoma. CSPG4 directed therapies might be particularly attractive in cases of high-grade conventional chondrosarcoma, local recurrence, and metastasis. Our study has 2 main limitations due to use of paraffin embedded tumors from one hospital and our three-tier system scoring approach for stained tumors that may have caused us to miss subtle differences in staining patterns. In addition the sample size in the survival analysis is small, especially for the dedifferentiated chondrosarcoma, limiting the possibility to account for potential confounders. Future studies might investigate CSPG4 protein expression in an independent patient series determining whether our data are generalizable. Nonetheless, our findings are consistent with published reports on CSPG4 as a prognostic marker in cancer and provide a rationale for future studies to investigate the effectiveness of CSPG4 directed therapy in chondrosarcoma *in vivo*.

## Data availability statement

The raw data supporting the conclusions of this article will be made available by the authors, without undue reservation.

## Ethics statement

The studies involving human participants were reviewed and approved by This study was approved by the Institutional Review Board at Massachusetts General Hospital (IRB approvalnumber: 2013P001012). Written informed consent for participation was not required for this study in accordance with the national legislation and the institutional requirements.

## Author contributions

All authors contributed to the study conception and design. Material preparation, data collection and analysis were performed by SN and DO-H. The first draft of the manuscript was written by SN and all authors commented on previous versions of the manuscript. All authors read and approved the final manuscript.

## Acknowledgments

This work was supported by National Institutes of Health grant RO1DE028172.

## Conflict of interest

The authors declare that the research was conducted in the absence of any commercial or financial relationships that could be construed as a potential conflict of interest.

## Publisher’s note

All claims expressed in this article are solely those of the authors and do not necessarily represent those of their affiliated organizations, or those of the publisher, the editors and the reviewers. Any product that may be evaluated in this article, or claim that may be made by its manufacturer, is not guaranteed or endorsed by the publisher.
