# Supplementary material for: Chondroitin sulfate proteoglycan 4 expression in chondrosarcoma: A potential target for antibody-based immunotherapy
Source: Front Oncol. 2022 Aug 30;12:939166. doi: 10.3389/fonc.2022.939166 (PMC9468862; doi:10.3389/fonc.2022.939166)
Supplement: Supplementary file 1 [file DataSheet_1.pdf]

10 Gy radiation increases the expression of CSPG4 on chondrosarcoma cell lines (CS1 and SW1353)

CS1

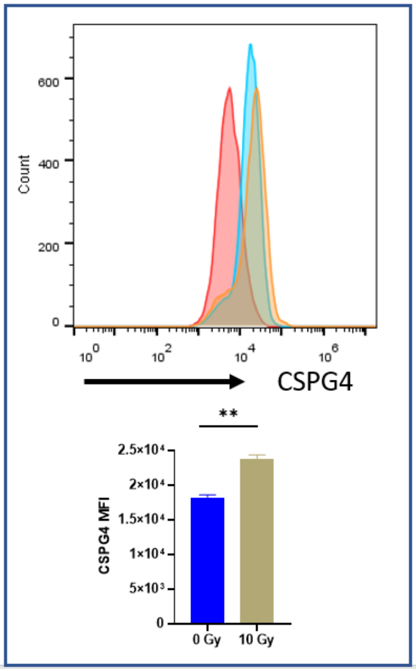

Isotype (MK2-23)

0 Gy

10 Gy

Day 0: Seed tumor cells at $5\text{x}10^5$ per well into 6 well plate
Day 1: 10 Gy radiation
Day 2: check CSPG4 expression via flow cytometry

SW1353

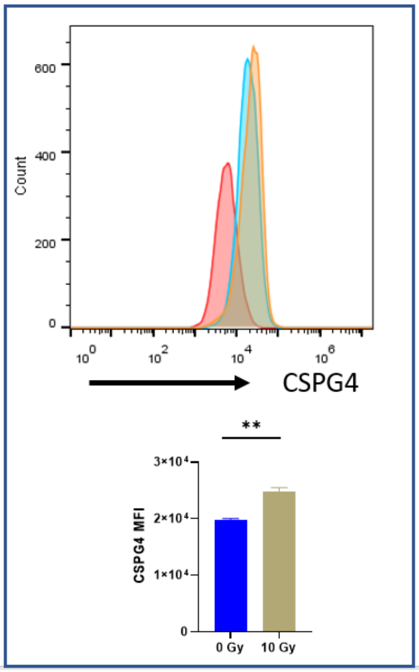

David Drum
1/24/22
